# Inflammatory and Oxidative Responses Induced by Exposure to Commonly Used e-Cigarette Flavoring Chemicals and Flavored e-Liquids without Nicotine

**DOI:** 10.3389/fphys.2017.01130

**Published:** 2018-01-11

**Authors:** Thivanka Muthumalage, Melanie Prinz, Kwadwo O. Ansah, Janice Gerloff, Isaac K. Sundar, Irfan Rahman

**Affiliations:** Department of Environmental Medicine, University of Rochester Medical Center, Rochester, NY, United States

**Keywords:** cigarettes, flavors, interleukin-8, monocytes, oxidative stress, inflammation, e-liquids

## Abstract

**Background:** The respiratory health effects of inhalation exposure to e-cigarette flavoring chemicals are not well understood. We focused our study on the immuno-toxicological and the oxidative stress effects by these e-cigarette flavoring chemicals on two types of human monocytic cell lines, Mono Mac 6 (MM6) and U937. The potential to cause oxidative stress by these flavoring chemicals was assessed by measuring the production of reactive oxygen species (ROS). We hypothesized that the flavoring chemicals used in e-juices/e-liquids induce an inflammatory response, cellular toxicity, and ROS production.

**Methods:** Two monocytic cell types, MM6 and U937 were exposed to commonly used e-cigarette flavoring chemicals; diacetyl, cinnamaldehyde, acetoin, pentanedione, o-vanillin, maltol and coumarin at different doses between 10 and 1,000 μM. Cell viability and the concentrations of the secreted inflammatory cytokine interleukin 8 (IL-8) were measured in the conditioned media. Cell-free ROS produced by these commonly used flavoring chemicals were also measured using a 2′,7′dichlorofluorescein diacetate probe. These DCF fluorescence data were expressed as hydrogen peroxide (H_2_O_2_) equivalents. Cytotoxicity due to the exposure to selected e-liquids was assessed by cell viability and the IL-8 inflammatory cytokine response in the conditioned media.

**Results:** Treatment of the cells with flavoring chemicals and flavored e-liquid without nicotine caused cytotoxicity dose-dependently. The exposed monocytic cells secreted interleukin 8 (IL-8) chemokine in a dose-dependent manner compared to the unexposed cell groups depicting a biologically significant inflammatory response. The measurement of cell-free ROS by the flavoring chemicals and e-liquids showed significantly increased levels of H_2_O_2_ equivalents in a dose-dependent manner compared to the control reagents. Mixing a variety of flavors resulted in greater cytotoxicity and cell-free ROS levels compared to the treatments with individual flavors, suggesting that mixing of multiple flavors of e-liquids are more harmful to the users.

**Conclusions:** Our data suggest that the flavorings used in e-juices can trigger an inflammatory response in monocytes, mediated by ROS production, providing insights into potential pulmonary toxicity and tissue damage in e-cigarette users.

## Introduction

E-cigarettes are gaining popularity among American youth mainly due to the availability of over 500 brands with over 7,700 uniquely flavored e-juices (Zhu et al., 2014). These flavoring chemicals are often generally recognized as safe (GRAS) classification when used in foods. E-cigarette consumption has been vastly increased over the recent years especially among American youth primarily due to flavors that are marketed with alluring names (Farley et al., 2014; Ambrose et al., 2015). With the declined consumption of cigarettes, e-cigarettes are advertised as a healthier alternative as the flavoring used in e-cigarettes are considered safe for ingestion (Berg et al., 2014; Klager et al., 2017). E-cigarette use has increased among adolescents, and the number of non-cigarette smoking youth who use e-cigarettes has tripled over the past years. This has become a serious public health concern as the non-smoking youth is twice as likely to consume conventional cigarettes (Bunnell et al., 2015; White et al., 2015). Moreover, some of the flavors used in e-liquids pose a potential health risk for its users (Allen et al., 2016; Kosmider et al., 2016; Gerloff et al., 2017).

Electronic nicotine delivery systems (ENDS), commonly known as e-cigarette is a battery-powered device that contains aerosolized nicotine delivered to its users in the form of vapor instead of smoke. It is assumed that e-cigarettes do not cause lung related diseases from toxic tobacco since e-cigarettes lack the combustion of tobacco. Therefore, it is generally thought that the effects of e-cigarettes are relatively less harmful than that of conventional cigarettes. However, the use of the e-cigarette should not be taken lightly because it has been on the United States market for only 10 years and more research needs to be done on e-cigarette constituents and their potential health effects. At present, e-liquids, cartridges and other vape products undergo minimal regulation under the Food and Drug Administration, FDA (Hutzler et al., 2014). E-liquids contain propylene glycol, nicotine and flavoring chemicals including diacetyl, cinnamaldehyde, acetoin, maltol, and pentanedione and other flavors including flavor enhancing chemicals (Allen et al., 2016). E-liquids come in a myriad of flavors at various nicotine concentrations ranging from 0 mg to 36 mg/mL (Davis et al., 2015). However, e-liquid constituents and their potential adverse effects have not been well-understood, and there is much scientific uncertainty about these products postulating an unrecognized respiratory health hazard to the users (Barrington-Trimis et al., 2014). In this study, we have only focused on the nicotine-free e-juices, as the effects and the mechanisms of nicotine are well established. These e-liquids can be categorized based on the flavor profile of the e-liquid. The categories include alcohol, berry, cake, candy, coffee/tea, fruit, menthol and tobacco (Table 1). Some of these flavors are pineapple coconut, cherry, cinnamon roll, café latte, cotton candy, melon, and tobacco.

**Table 1:** Categorization of e-liquids by flavor^*^.

**Alcohol**	**Berry**	**Cake**	**Candy**	**Coffee/Tea**	**Fruit**	**Menthol**	**Tobacco**
Pineapple Coconut (Ecto)	Cherry (Smoker's Choice Rochester)	Apple Pie (Ecto)	Sweet Fishies (Ecto)	Cafe Royale (Cyber Liquids)	Mega Melons (Cuttwood)	Mystery Mix (Ecto)	American Tobacco (Ecto)
	Strawberry (Smoker's Choice Rochester)	Banana Nut Bread (Ecto)	Fruit Swirl (Ecto)	Cafe Latte (Ecto)	Tangerine (Smoker's Choice Rochester)		Classic Tobacco (Vape Dudes)
	Cherry (Ecto)	Cinnamon Roll (Vape Dudes)	Cotton Candy (Vape Dudes)	Chai Tea (Ecto)	Grape Vape (Vape Dudes)		Marbo (Upstate Vape)
	Very Berry (Vapor Drops)		Orange Creamsicle (Ecto)		Peaches N Cream (Drip)		9X Tobacco (Upstate Vape)
	Strawberry Fields (Vape Dudes)		Grape Jam (Vape Jam)		Pineapple Express (Drip)		Tobacco (Vapor Drops)
	Strawberry Zing (Vape Dudes)		Bird Brains (Cuttwood)		Melon Mania (Drip)		
	Berry Intense (Drip)		Euphoria (Cosmic Fog)		Peach (Ecto)		
					Plasma (Ecto)		

* *E-liquids were obtained from vendors and categorized according to the flavor*.

The e-liquid manufacturers market these liquids with alluring names, such as Cotton Candy, Oatmeal Cookie, and Tutti Frutti that are more appealing especially to young adults (Allen et al., 2016). Vaping exposes these flavoring chemicals to the lungs when the e-liquids are heated and inhaled with a similar mechanistic pathway as the inhalation of chemicals at microwave popcorn factories and coffee roasting plants (Bailey et al., 2015).

The flavors used in e-cigarettes are known to cause inflammatory and oxidative stress responses in lung cells (Baggiolini and Clark-Lewis, 1992; Aw, 1999; Lerner et al., 2015b; Gerloff et al., 2017). In this study, we assessed the inflammatory response of monocytic cells due to the exposure of nicotine-free e-liquid flavors and commonly used e-liquid flavoring chemicals, such as diacetyl, cinnamaldehyde, pentanedione, acetoin, maltol, ortho-vanillin, and coumarin. We assessed inflammation by quantifying interleukin 8 (IL-8), a major pro-inflammatory marker primarily produced by macrophages involved in neutrophil recruitment during inflammation (Moldoveanu et al., 2009). The potential to cause oxidative stress by these flavoring chemicals and e-liquids were assessed by cell-free reactive oxygen species (ROS) assay. We hypothesized that the inflammatory response due to the acute exposure of e-liquids and flavoring chemicals is mediated by oxidative stress and these responses are dose-dependent.

## Materials and methods

### Scientific rigor

We used rigorous and unbiased approach during experiments and data analysis.

### Classification of e-Liquid and flavors

We have classified the e-liquid based on their flavor characteristics (Table 1).

### Culturing U937 and mono mac 6 (MM6) cells

U937 monocytic cells from human pleural tissue were obtained from ATCC. Cells were cultured and grown to reach the required density in complete RPMI 1640 medium with 5% FBS and 1% penicillin/streptomycin in T75 flasks. Passages below 10 were selected and seeded at 500,000 cells per well in 24 well plates with 1 ml of complete RPMI 1640 media with 1% FBS. After incubating the cells overnight, they were treated with flavoring chemicals or flavored e-liquids.

The human monocyte-macrophage cell line (mature monocytes-macrophages) Mono Mac 6, which was established from peripheral blood of a patient with monoblastic leukemia were grown in RPMI 1640 medium supplemented with 10% FBS, 2 mM l-glutamine, 100 μg/ml penicillin, 100 U/ml streptomycin, 1% nonessential amino acids, 1 mM sodium pyruvate, 1 μg/ml human holo-transferrin, and 1 mM oxaloacetic acid. The cells were cultured at 37°C in a humidified atmosphere containing 5% CO_2_. When the sufficient density was reached, the cells were seeded in 6-well plates at the density of 1 × 10^6^ cells in 2 ml supplemented media with 1% FBS and incubated at 37°C with 5% CO_2_ overnight, prior to the exposure of the cells to flavoring chemicals or e-liquids. Cells were incubated in low serum containing media (FBS 1%) to reduce unwanted stimulation of the cells and the background cytokine levels. Serum starvation allowed us to measure subtle changes in cytokine level due to the treatment of interest.

### Cell treatments and collection of conditioned media

Serum-deprived U937 and MM6 cells were treated with flavoring chemicals diacetyl, cinnamaldehyde, acetoin, maltol, pentanedione, o-vanillin, and coumarin. Each flavoring chemical was added to designated wells at varying concentrations between 10 and 1,000 μM in triplicates. This wide range of concentration was chosen based on our earlier publication (Gerloff et al., 2017) and on the notion to assess the elicited inflammatory/oxidative stress response by macrophages with minimum cellular toxicity. Twenty-four hours post-treatment, the conditioned media was collected by centrifugation of MM6 cell suspension at 1,000 rpm for 5 min and U937 cell suspension at 125 g for 7 min. Collected supernatants were frozen at −80°C for cytokine assessment. The viability of the cells was measured by re-suspending the cells in PBS.

U937 cells were also treated with a selected number of flavored e-liquids without nicotine at 0.25 and 0.5% concentrations. The flavored e-liquids used for treatments included Strawberry Zing, Café Latte, Pineapple Coconut, Cinnamon Roll, Fruit Swirl, Mega Melons, Mystery Mix (menthol flavor), American Tobacco, Grape Vape, Very Berry, and Mixed Flavors (an equally proportional mixture of the e-liquids). Untreated and propylene glycol treated cell groups served as the control and the solvent control groups.

### Cytotoxicity via cell viability assessment

Using the acridine orange (AO) and propidium iodide (PI) staining, viability was determined in U937 and MM6 cells for plating and after treatment with flavoring chemicals and e-liquids. AO/PI staining and viability determination was performed in 20 μL of cells combined with 20 μL of AO/PI staining solution. Finally, 20 μL of stained cells were then added to a Cellometer counting chamber and analyzed using a fluorescent Cellometer (Nexcelom Bioscience, Lawrence MA). At the end of the analysis, the Cellometer automatically reported live and dead cell concentration as a percentage.

### Cell-free ROS assay for flavoring chemicals and flavored e-Liquids

The relative levels of OX/ROS produced from flavoring chemicals or e-cig vapor were determined using 2′,7′dichlorofluorescein diacetate (H2 DCF-DA) fluorogenic probe (EMD Bioscience, CA). A spectrofluorometer (Turner Quantech fluorometer Model FM109535 from Barnstead International/Thermolyne Corporation) was used to measure oxidized dichlorofluorescein (DCF) fluorescence at absorbance/emission maxima of 485 nm/535 nm. Hydrogen peroxide standards between 0 and 50 μM were created from 1 M stock and reacted at room temperature for 10 min with the prepared DCFH solution in a total of 5 ml. These standards were then used to calibrate fluorescence intensity units (FIU) which numerically match respective hydrogen peroxide (H_2_O_2_) concentrations. Flavoring chemical concentrations for acetoin, diacetyl, 2′,3′ pentanedione, cinnamaldehyde, maltol, o-vanillin, and coumarin between 10 and 1,000 μM were prepared in phosphate buffer. After mixing the dye with the flavoring chemical and incubating at 37°C for 15 min, the fluorescence was recorded for each flavoring chemical. The DCF fluorescence data are expressed as μM H_2_O_2_ equivalents referring to the concentration of the H_2_O_2_ added to the DCFH solution.

To assess the ROS with a new atomizer, flavored e-liquids from Table 1 (Strawberry Zing, Strawberry Fields, Very Berry, Grape Vape, American Tobacco, Mystery Mix, and Mixed Flavors) were aerosolized with a new atomizer at each use using the Scireq inExpose (Montreal, Canada) e-cigarette system with one puff per minute for 10 minutes. “Mixed Flavors” were prepared by combining an equal amount of each of the selected flavored e-liquid (Strawberry Zing, Café Latte, Pineapple Coconut, Cinnamon Roll, Fruit Swirl, Mega Melons, Mystery Mix (menthol flavor), American Tobacco, Grape Vape and Very Berry) together. Subsequently, aerosol from flavored e-liquid was bubbled through the DCFH solution at 60 L/min. The bubbled DCF solution was then measured for ROS release.

To obtain ROS values with a used atomizer, selected e-liquids from Table 1 (Café Latte, Cinnamon Roll, Chai tea, Pineapple Coconut, and Cotton Candy) were aerosolized with a previously used atomizer using the Scireq inExpose e-cigarette system as described above. In between switching different flavors, propylene glycol was aerosolized for 10 min. This exemplifies the concept of attempting to clean the atomizer in order to avoid residual carryover from one e-liquid flavor to the next. E-liquid flavor aerosol was bubbled through the DCFH solution at 60 L/min. The bubbled DCF solution was then measured for ROS release. Propylene glycol (PG) was used as a control comparison group.

To obtain cell-free ROS assay for “consecutive flavors,” 10 flavored e-liquids (Strawberry Zing, Café Latte, Pineapple Coconut, Cinnamon Roll, Fruit Swirl, Mega Melons, Mystery Mix (menthol flavor), American Tobacco, Grape Vape, and Very Berry) were aerosolized two puffs per e-liquid flavor, one flavor at a time for 10 min. Flavored e-liquid aerosols were bubbled through the DCFH solution and then measured for ROS release. Propylene glycol (PG) was used as a control when measuring ROS release.

### Inflammatory response (IL-8) assay

Following cell treatments, conditioned media were collected 24 h post-treatment of different concentrations of flavoring chemicals. Pro-inflammatory cytokine (IL-8) release was determined using the IL-8 cytoset ELISA kit according to the manufacturer's instructions (Life Technologies).

### Statistical analysis

Statistical analyses of significance were performed by one-way ANOVA (Tukey's multiple comparison test) when comparing multiple groups and student *t*-test when comparing two groups using GraphPad Prism 7 (La Jolla, CA). Data are presented as means ± SEM. *P* < 0.05 is considered as statistically significant.

## Results

### Cytotoxicity due to flavoring chemicals

**Figure 1:**
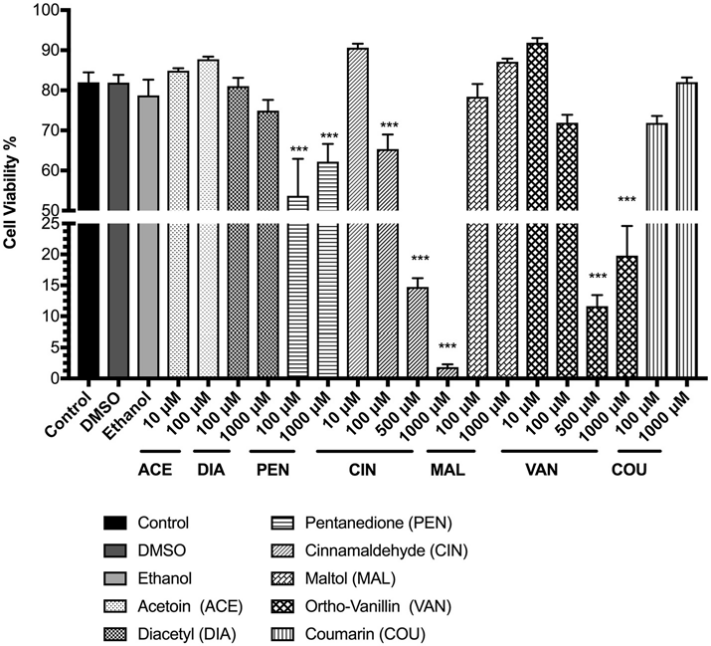
Percent viability of U937 cells 24 h post-exposure to e-cigarette flavoring chemicals, i.e., acetoin, diacetyl, pentanedione, cinnamaldehyde, maltol, o-vanillin, and coumarin at concentrations between 10 μM and 1,000 μM. U937 monocytes were treated with e-cigarette flavoring chemicals at varying concentrations and incubated at 37°C with 5% CO_2_ for 24 h. Cells were rinsed with PBS and stained with AO/PI dye. The viability of the cells was assessed using the Cellometer 2000. Data are expressed as mean ± SEM (*n* = minimum 3 per group). Statistical significance was determined by one-way ANOVA (Tukey's multiple comparison test). ^***^p < 0.001 vs. Control.

To assess the cytotoxicity due to exposure to flavoring chemicals U937 and MM6 cells were stained with AO/PI dye after 24 h. In U937 cells, flavoring chemical treatments with 2, 3-pentanedione, cinnamaldehyde, and o-vanillin significantly affected the cell viability compared to the untreated control group (Figure 1). Pentanedione treatment reduced the cell viability to about 62% (*p* < 0.001). Cinnamaldehyde treatment showed a distinct dose-dependent cytotoxic response, decreasing the cell viability to 65, 15, and 2% with 100, 500, and 1,000 μM concentrations respectively (*p* < 0.001). Treatment with o-vanillin reduced the cell viability to approximately between 12 and 19% (*p* < 0.001). Other flavoring chemicals, acetoin, diacetyl, maltol, and coumarin did not affect the cell viability at the tested concentrations. To assess any effects on viability by the solvents used with the flavoring chemicals, DMSO and ethanol treatments were also performed in which no considerable effects on cell viability were observed.

**Figure 2:**
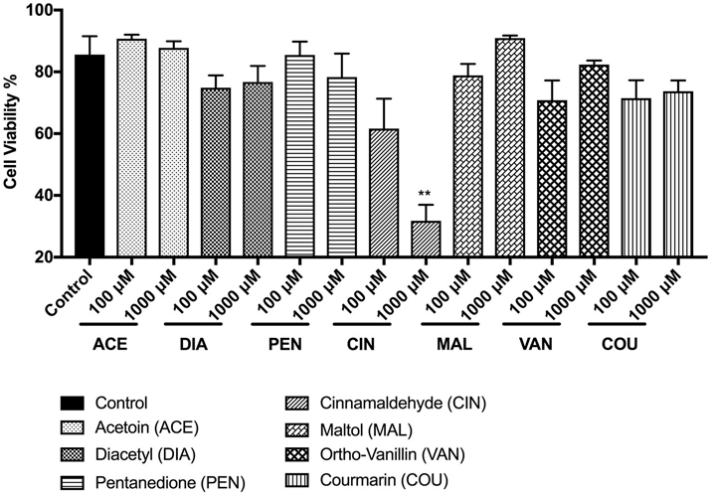
Percent viability of Mono Mac 6 (MM6) cells 24 h post-exposure to e-cigarette flavoring chemicals, i.e., acetoin, diacetyl, pentanedione, cinnamaldehyde, maltol, o-vanillin, and coumarin at concentrations 100 and 1,000 μM. Mono Mac 6 cells were treated with e-cigarette flavoring chemicals and incubated at 37°C with 5% CO_2_ for 24 h. Cells were rinsed with PBS and stained with AO/PI dye. The viability of the cells was assessed using the Cellometer 2000. Data are expressed as mean ± SEM (*n* = 3 per group). Statistical significance was determined by one-way ANOVA (Tukey's multiple comparison test). ^**^p < 0.01 vs. Control.

In MM6 cells, the tested flavoring chemicals caused no significant cell death except in cinnamaldehyde treatment groups (Figure 2). The cell viability of the other treated groups; acetoin, diacetyl, pentanedione, maltol, vanillin, and coumarin ranged above 70%. At 100 and 1,000 μM cinnamaldehyde concentrations, MM6 cell viability was reduced to 61 and 32% respectively (Figure 2). Only with the cinnamaldehyde treatment, we observed a dose-dependent cytotoxic response (*p* < 0.01) compared to the untreated control group.

### Cytotoxicity due to flavored e-Liquid exposure

**Figure 3:**
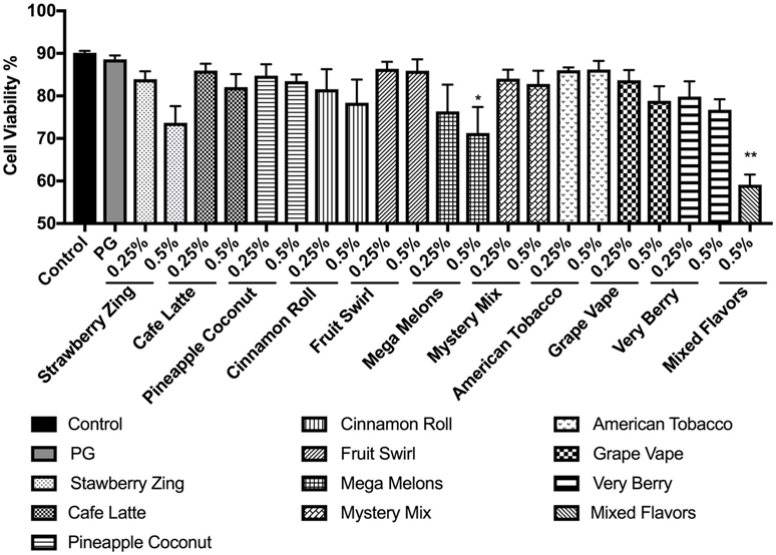
Percent viability of U937 cells 24 h post-exposure to e-liquid base propylene glycol and selected nicotine-free e-liquids, i.e., Strawberry Zing, Café Latte, Pineapple Coconut, Cinnamon Roll, Fruit Swirl, Mega Melons, Mystery Mix, American Tobacco, Grape Vape, Very Berry, and mixed flavors at two concentrations 0.25% and 0.5%. U937 monocytes were treated with e-liquids at two concentrations, 0.25% and 0.5% (mixed e-liquid treatment only at 0.5%) for 24 h. Cells were then rinsed with PBS and stained with AO/PI. The viability of the cells was assessed using the Cellometer 2000. Data are expressed as mean ± SEM (*n* = 5 per treatment group). Statistical significance was determined by one-way ANOVA (Tukey's multiple comparison test). ^*^p < 0.05, ^**^p < 0.01 vs. control.

In order to assess the cytotoxicity of the flavored e-liquids, we exposed U937 cells to 0.25 and 0.5% concentrations of selected e-liquids from Table 1. Typically, e-liquid base includes propylene glycol (PG). Thus, PG was used as a control. PG showed no cytotoxicity. Tested e-liquids caused decreased cell viability at the higher dose for each e-liquid in general. However, only Mystery Mix exhibited significant cytotoxicity, reducing cell viability to 71% (*p* < 0.05). Treating the cells with “mixed flavors” e-liquids at 0.5% concentration decreased the cell viability to 59% (*p* < 0.01) (Figure 3).

### Cell-free ROS release by flavoring chemicals and with flavored e-Liquids

**Figure 4:**
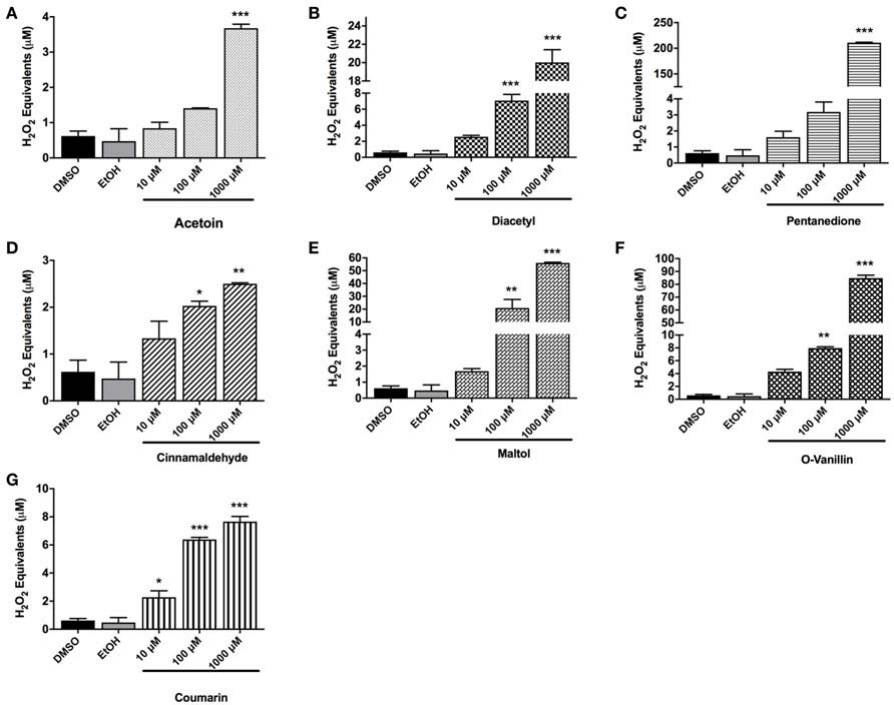
Cell-free ROS in flavoring chemicals. **(A)** Acetoin, **(B)** diacetyl, **(C)** pentanedione, **(D)** cinnamaldehyde, **(E)** maltol, **(F)** o-vanillin, and **(G)** coumarin flavoring chemicals were added to DCFH OX/ROS indicator solution at 10 μM, 100 μM, and 1,000 μM concentrations. Oxidized DCF fluorescence was measured using a fluorometer. Data are shown as mean ± SEM (*n* = 2–3 per group). Statistical significance was determined by one-way ANOVA statistical analysis (Tukey's multiple comparisons test). ^*^P < 0.05, ^**^P < 0.01, ^***^P < 0.001, vs. DMSO and EtOH.

To measure the amount of exogenous ROS released by flavoring chemicals in e-liquids, the DCFH-DA dye was treated with the flavoring chemicals of interest, and the florescence was measured. The concentration of the ROS was expressed as H_2_O_2_ equivalents. For all the tested flavoring chemicals, acetoin, diacetyl, pentanedione, cinnamaldehyde, maltol, o-vanillin, and coumarin, the solvent controls (DMSO and ethanol) gave rise to extremely low H_2_O_2_ equivalents. For all the chemicals, the H_2_O_2_ equivalents at 10 μM concentration were minimal, whereas at 1,000 μM concentration it was significantly elevated (*p* < 0.001) compared to control DMSO and EtOH. Diacetyl, cinnamaldehyde, maltol, and o-vanillin significantly elevated H_2_O_2_ equivalents at 100 μM concentration. While acetoin, diacetyl, pentanedione, cinnamaldehyde, maltol and o-vanillin exhibited moderately increased ROS levels at 10 μM concentration, only coumarin showed a significant increase in ROS levels compared to the control groups (*p* < 0.05) (Figures 4A–G).

**Figure 5:**
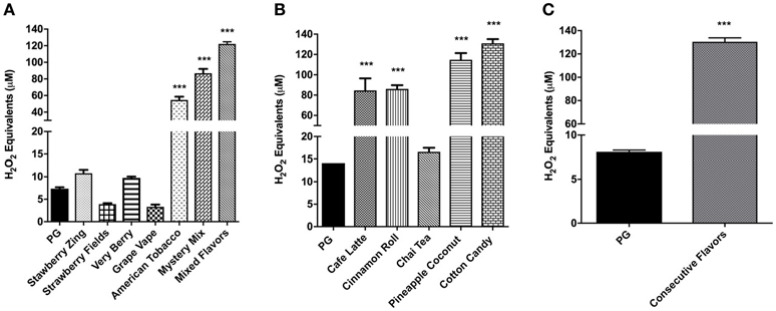
**(A)** Cell-free ROS in flavored e-liquids with a new atomizer at each use with one puff per min. E-liquids (Strawberry Zing, Strawberry Fields, Very Berry, Grape Vape, American Tobacco, Mystery Mix and Mixed Flavors) aerosols were drawn through the DCFH solution using a SciReq inExpose. Oxidized DCF fluorescence was measured using a fluorometer. Data are shown as mean ± SEM (*n* = 6 per group). Statistical significance was determined by One-way ANOVA (Tukey's multiple comparison test). ^***^P < 0.001 vs. propylene glycol. **(B)** Cell-free ROS in selected e-liquids using a PG aerosolized atomizer. Selected e-liquid aerosols (Café Latte, Cinnamon Roll, Chai Tea, Pineapple Coconut and Cotton Candy) were aerosolized using a SciReq inExpose and drawn through DCFH with PG aerosolization in between e-liquids. Oxidized DCF fluorescence was measured using a fluorometer. Data are shown as mean ± SEM (*n* = 2–6 per group). Statistical significance was determined by One-way ANOVA (Tukey's multiple comparison test). ^***^p < 0.0001 vs. propylene glycol. **(C)** Cell-free ROS in acute exposure of consecutively aerosolized flavors. Ten e-liquid flavors (Strawberry Zing, Café Latte, Pineapple Coconut, Cinnamon Roll, Fruit Swirl, Mega Melons, Mystery Mix, American Tobacco, Grape Vape and Very Berry) were aerosolized consecutively (consecutive mixture of flavors) using a SciReq inExpose machine one flavor at a time during a cumulative 10 min period and drawn through DCFH. Oxidized DCF fluorescence was measured using a fluorometer. Data are shown as mean ± SEM (*n* = 6). Statistical significance was determined by student *t*-test. ^***^p < 0.001 vs. PG.

To measure the cell-free OX/ROS produced by flavored e-liquids with a new atomizer, the aerosols were bubbled through the DCF-DA indicator solution, then the fluorescence was measured as H_2_O_2_ equivalents. As shown in Figure 5A, Strawberry Zing, Very Berry, American Tobacco, Mystery Mix, and Mixed Flavors produced higher H_2_O_2_ equivalents compared to PG (*p* < 0.001). Respectively, American Tobacco, Mystery Mix, and Mixed Flavors had the highest H_2_O_2_ equivalents compared to PG (*p* < 0.001) (Figure 5A).

In order to quantify the ROS levels released with a used atomizer, the same atomizer was continuously used with selected e-liquids and PG was used in between to reduce the carryover of residual ROS from one e-liquid to the next during aerosolization. While Chai Tea produced comparable H_2_O_2_ equivalents to PG, Café Latte, Cinnamon Roll, and Cotton Candy produced highly significant levels of H_2_O_2_ equivalents compared to the control PG group (*p* < 0.001) (Figure 5B).

### Cell-free ROS release by consecutive mixture of flavors

Consecutive aerosolization of 10 different e-liquids produced significantly elevated H_2_O_2_ equivalents compared to the control PG (*p* < 0.001) (Figure 5C). This OX/ROS amount was comparable to the Mixed Flavors in Figure 5A.

### Inflammatory mediator (IL-8) response due to flavoring chemicals

The inflammatory response due to the exposure to flavoring chemicals was assessed by treating MM6 and U937 monocytic cells with flavoring chemicals and measuring the IL-8 concentrations in the conditioned media.

**Figure 6:**
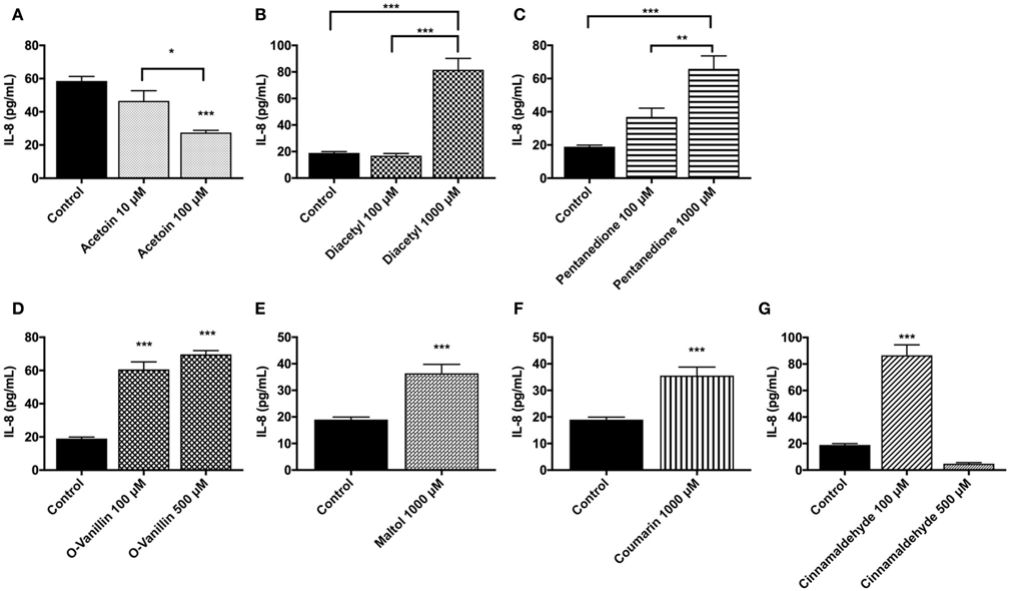
Flavoring chemicals, **(A)** acetoin, **(B)** diacetyl, **(C)** pentanedione, **(D)** o-vanillin, **(E)** maltol **(F)** coumarin, and **(G)** cinnamaldehyde (at low and high doses between 10 and 1,000 μM) induced pro-inflammatory cytokine, IL-8, response by U937 cells. U937 monocytes were treated with flavoring chemicals for 24 h. Conditioned media was then assayed for IL-8 concentration by ELISA. Data are expressed as mean ± SEM. *N* = 4–6 per group. Statistical significance was determined by One-way ANOVA for multiple groups (Tukey's comparisons test). ^*^p < 0.05, ^**^p < 0.01, ^***^p < 0.001 vs. untreated control. Student *t*-test for comparing two groups. ^***^p < 0.001 vs. untreated control.

In U937 cells, treatment with flavoring chemicals of interest was performed at least twice with various dose concentrations. Representative treatment and its respective control data sets were chosen. Treatment with acetoin decreased IL-8 levels in a dose-dependent manner. At 1,000 μM concentration, this downregulation in IL-8 cytokine is highly significant (*p* < 0.0001) (Figure 6A). Treatment with a concentration of 1,000 μM diacetyl resulted in a significant elevation in IL-8 levels (*p* < 0.0001) (Figure 6B). 2, 3-Pentanedione and o-vanillin treatments caused a significant increase in IL-8 response in a dose-dependent manner (Figures 6C,D). Maltol and coumarin treated groups (1,000 μM concentration) increased the IL-8 concentrations significantly (*p* < 0.001) (Figures 6E,F). Treatment with 10 μM concentration of cinnamaldehyde increased the IL-8 highly significantly (*p* < 0.001), whereas 1,000 μM concentration of cinnamaldehyde treatment reduced the IL-8 lower than its untreated control likely due to the cytotoxicity of the treatment (Figure 6G).

In MM6 cells, acetoin, cinnamaldehyde, and vanillin showed increased IL-8 responses compared to the untreated control group (% increase vs. controls: acetoin 100 μM concentration = 54.4% and 1,000 μM concentration = 78.7%; cinnamaldehyde 1,000 μM concentration = 72.2%; vanillin 100 μM concentration = 107.1% and 1,000 μM concentration = 31.1%). Diacetyl and coumarin treatments did not show an appreciable increase in IL-8 release in the treated groups in comparison to the untreated control group (data not shown).

### Inflammatory response (IL-8) due to flavored e-Liquid exposure

**Figure 7:**
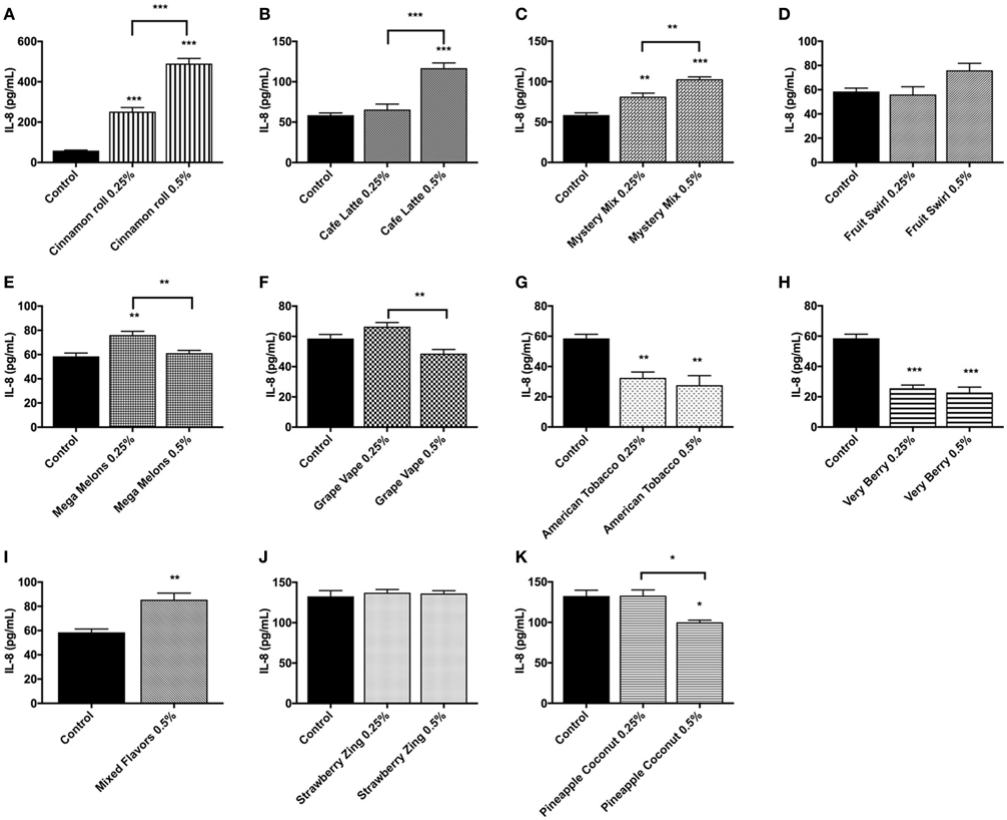
E-liquids **(A)** Cinnamon Roll, **(B)** Café Latte, **(C)** Mystery Mix, **(D)** Fruit Swirl, **(E)** Mega Melons, **(F)** Grape Vape, **(G)** American Tobacco, **(H)** Very Berry, **(I)** Mixed Flavors, **(J)** Strawberry Zing, and **(K)** Pineapple Coconut induced pro-inflammatory cytokine, IL-8, response by U937 cells. U937 monocytes were treated with e-liquids at two doses, 0.25% and 0.5%, for 24 h. Conditioned media was then assayed for IL-8 concentration by ELISA. Data are expressed as mean ± SEM. (*N* = 4 per group. Statistical significance was determined by One-way ANOVA for multiple groups (Tukey's comparisons test). ^*^p < 0.05, ^**^p < 0.01, ^***^p < 0.001 vs. untreated control. Student *t*-test for comparing two groups (^**^p < 0.01 and ^***^p < 0.001 vs. untreated control).

Inflammatory response due to flavored e-liquid treatment was assessed by the measurement of IL-8 concentrations in conditioned media after 24 h of flavored e-liquid treatment. These treatments were performed twice, and representative data sets were chosen with its corresponding control. The untreated control cells had relatively low IL-8 levels compared to the treated groups, in most cases averaging around 50 pg/mL. Cinnamon Roll and Mystery Mix showed significant dose-dependently increasing levels of IL-8 with *p* < 0.01 or stronger at either dose (Figures 7A,C). Café Latte and Mixed Flavors e-liquid treatment at 0.5% caused a highly significant IL-8 response (*p* < 0.001) (Figures 7B,I). Interestingly, treatment with Mega Melons, Grape Vape, and Pineapple Coconut either had a slight increase or equal levels of IL-8 at 0.25% dose and a significant decrease in IL-8 levels at 0.5% dose compared to their untreated counterparts (Figures 7E,F,K). Similarly, treatment with American Tobacco and Very Berry significantly reduced the IL-8 response even at 0.25% dose (Figures 7G,H). Treatment with Fruit Swirl and Strawberry Zing had comparable IL-8 levels to the untreated control (Figures 7D,J).

## Discussion

E-cigarettes hold the popular misconception that they have relatively less or no harm to the consumer's health in contrast to conventional combustible tobacco due to lack of sufficient evidence to prove its harmful effects. These uncertainties are primarily due to many unstandardized facets of ENDS such as e-liquid constituents and unstandardized e-cigarette devices. Many studies have shown that the consumption of e-cigarettes potentially causes harm to pulmonary, cardiovascular, immune and nervous systems (Qasim et al., 2017). The adverse health effects of nicotine have been well established; however, health effects related to e-cigarettes without nicotine are still emerging. These health effects are mainly due to constituents of e-liquid vapors (Varlet et al., 2015). Studies have shown that e-liquid aerosols contain significant levels of toxic compounds, such as aldehydes and acrolein that are detrimental to e-cigarette users (Sleiman et al., 2016; Talih et al., 2016).

The focus of this study was to investigate the oxidative stress and inflammatory effects of commonly used e-cigarette flavoring chemicals and flavored e-liquids without nicotine. We selected cell-free ROS levels and IL-8 levels as they are well established biomarkers for oxidative stress mediated inflammation and tissue damage (Vlahopoulos et al., 1999; Mittal et al., 2014; Lerner et al., 2015b). Exogenous ROS levels produced by flavoring chemicals and e-liquids were quantified in this study. Oxidative stress caused by these reactive species activates inflammatory genes, such as IL-8 chemokine. IL-8 has a profound effect on neutrophil recruitment and activation. We have previously demonstrated that the exposure to e-cigarette flavoring chemicals induces a significant IL-8 response (Lerner et al., 2015b; Gerloff et al., 2017).

The flavoring chemicals, acetoin, diacetyl, pentanedione, cinnamaldehyde, maltol, ortho-vanillin, and coumarin were tested in this study. According to Tierney et al., e-liquids contain 10–40 mg/mL of total flavoring chemicals (Tierney et al., 2016). Treatment concentrations from 10 to 1,000 μM were selected to encompass and account for the variability in consumption due to low voltage and high voltage ENDS and the vaping habits.

Among the flavoring chemicals tested, cinnamaldehyde showed the most toxicity to both the cell types. O-vanillin and pentanedione also showed significant cytotoxicity. These results are consistent with other studies that were recently published showing significant cytotoxicity of flavors such as “Cinnamon Ceylon” on various other cell lines such as epithelial cells and fibroblasts (Bahl et al., 2012; Behar et al., 2016). Treatment of cells with selected e-liquids from commonly marketed categories exhibited cytotoxicity. Mystery Mix, a selection from the “menthol” category, showed significant cytotoxicity. This is consistent with other *in vitro* studies in which other investigators have found significant cytotoxicity with menthol flavoring aerosol exposures on epithelial cell lines (Leigh et al., 2016; Singh et al., 2016). Mixing equal proportions of e-liquids from 10 differently flavored e-liquids gave rise to the highest cytotoxicity. This suggests that e-cigarette users who inhale a variety of flavored e-liquids at social events are perhaps prone to higher toxic effects than those who vape a single flavor of e-liquid.

The OX/ROS analysis revealed that all the flavoring chemicals of interest produced significant levels of H_2_O_2_ equivalents. Moreover, we observed that several e-liquids (American tobacco, Mystery Mix, Café Latte, Cinnamon Roll, Pineapple Coconut, and Cotton Candy) also produced significant amounts of H_2_O_2_ equivalents. There was no distinct trend in ROS release with a new or used atomizer suggesting that continuous use of an atomizer does not enhance the ROS production. Mixing various flavors of e-liquids together produced comparable H_2_O_2_ equivalents to aerosolizing the same e-liquid flavors consecutively. This simulates a social situation where smokers exchange and vape several e-liquid flavors in a short period of time. This data suggest that acute exposure to a combination of e-liquid flavors is more harmful than the exposure to a single flavor. This response is consistent with the cell viability and IL-8 data where exposure to Mixed Flavors was more cytotoxic compared to individual flavors and caused significant inflammation. The presence of ROS in e-liquids can potentially cause oxidative stress related lung injury and diseases such as asthma, bronchiectasis/bronchiolitis obliterans, COPD and pulmonary fibrosis (Park et al., 2009). This is consistent with the human study conducted by Carnevale et al., showing that the use of e-cigarettes increases oxidative stress/injury biomarkers, such as 8-isoprostanes in blood compared to non-smokers (Carnevale et al., 2016).

Pro-inflammatory cytokine, IL-8, is a neutrophil chemoattractant mediating the inflammatory process. IL-8 plays a crucial role in the pathogenesis of chronic inflammation and cancer (Mukaida, 2003). In our study, we observed that diacetyl, pentanedione, o-vanillin, maltol, coumarin, and cinnamaldehyde induced significant levels of IL-8 secretion in MM6 and U937 monocytes. This upregulation was also observed with several e-liquids, such as Cinnamon Roll, Café Latte, Mystery Mix, Mega Melons, and with Mixed Flavors. These findings are similar to other studies that showed an increased pro-inflammatory response in other cells, such as THP-1 monocytes and primary human airway epithelial cells (Wu et al., 2014; Ween et al., 2017). In contrast, with the acetoin treatment, we observed a dose-dependent reduction in IL-8 secretion. It may be due to immuno-suppressive effects, as there have been several studies with similar results, e.g., Clapp et al observed immunosuppression in alveolar macrophages and NK cells caused by cinnamaldehyde treatment (Clapp et al., 2017). Martin et al observed down-regulation of CSF-1 and CCL26 inflammatory genes (Martin et al., 2016). Reidel et al. found increased neutrophilic activation and mucin hypersecretion by e-cigarette in users (Reidel et al., 2017). Many studies have shown that e-cigarette exposure can dampen immunity against bacteria, such as *Streptococcus pneumonia, Staphylococcus aureus*, and viruses, such as influenza A in mice (Sussan et al., 2015; Hwang et al., 2016).

Our data suggest that the presence of ROS in flavored e-liquids could play an essential role in the oxidative stress-mediated inflammatory response. This is consistent with previous studies conducted by our laboratory on lung epithelial cells and C57BL/6 mice (Lerner et al., 2015b). It is possible that ROS initiate the activation of transcription factors, such as NF-κB, STAT3, AP-1, and Nrf2 resulting in the propagation of other cellular and inflammatory responses such as secreting inflammatory cytokines and regulating the antioxidant defense systems (Kreiss et al., 2002; Reuter et al., 2010; Morgan and Liu, 2011). Thus, IL-8 modulation in monocytes treated with flavored e-liquids and flavoring chemicals was observed.

Recent studies have demonstrated that the most preferred e-liquid flavors are the sweet, fruity, creamy, and buttery flavors. Zeng et al. also showed that there is a high frequency of mixing of those flavors together by the consumers during vaping (Kim et al., 2016; Chen and Zeng, 2017). These commonly consumed flavors are derived from flavoring chemicals tested in our study. The most prevalent class of compounds in e-liquids is aldehydes which include acetaldehyde and formaldehyde (example: vanilla flavor). Most prevalent non-aldehydes include acetoin and diacetyl (Klager et al., 2017; Ogunwale et al., 2017). The most prevalent alcoholic compound classes include alcohols, such as maltol and menthol (Tierney et al., 2016). Other most common flavoring chemicals include acetoin, diacetyl, and 2'3'-pentanedione (Allen et al., 2016). Obliterative bronchiolitis (bronchiolitis obliterans) is a disease caused by exposure to butter flavoring chemicals (diacetyl, 2, 3-pentanedione). Chronic inhalation of these chemicals causes airway epithelium injury ultimately resulting in the formation of pro-fibrotic lesions (Morgan et al., 2012; Flake and Morgan, 2017; Wallace, 2017). Chocolate flavoring chemical, 2.5-dimerthylpyrazine has shown to alter cystic fibrosis transmembrane conductance regulator (CFTR) expression, which could have adverse effects in immune mechanisms, such as mucociliary clearance, dampening the epithelial defense against inhaled particulates and pathogens (Sherwood and Boitano, 2016). Mucus-hypersecretion can hinder the respiratory pathogen clearance and exacerbate respiratory function in pulmonary diseases, such as COPD and asthma (Vareille et al., 2011). ROS present in flavoring chemicals and flavored e-liquids can also bind to biomolecules, such as DNA and cause adducts along with histone modifications (Sundar et al., 2016). Prior studies have shown that e-cigarettes release nanoparticles in comparable amounts to combustible cigarettes, which can deposit deep in the alveolar region to smaller airways/peripheral areas. Inhaling these nanoparticles provides a route of exposure of toxic chemicals to the bloodstream (Lee et al., 2017). These nanoparticles included copper, tin, chromium and nickel that can pose detrimental health risks (Williams et al., 2013; Lerner et al., 2015a). Findings in our study as well as from others imply that there is much to be scientifically investigated and the ENDS must be standardized. E-liquid flavoring chemicals and other constituents must be tightly regulated to minimize the risk of lung disease especially among teens.

There are several limitations to this study. Exposure of U937 monocytes directly to the e-liquid provided meaningful toxicological data. However, it ideally would be preferable to expose the cells to e-liquid aerosols with lower concentrations to understand the cellular toxicity of flavored e-liquid aerosol. As a future direction, we intend to perform *in vitro* and *in vivo* flavored e-liquid aerosol exposures and assess the inflammatory cytokine profile. Lastly, only one crucial chemokine/cytokine was measured in this study. We plan to quantify other inflammatory mediators induced by acute and chronic flavored e-liquid exposures in the future.

In conclusion, cinnamaldehyde, vanillin, and pentanedione were the most toxic flavoring chemicals on monocytes. Majority of the tested flavoring chemicals and the e-liquids caused the secretion of significantly elevated pro-inflammatory cytokine levels by monocytes. Mixing multiple flavors of e-liquids caused the greatest cytotoxicity implying the health risk of acute exposure to a variety of e-liquids as opposed to a single flavor. Some flavors and their key flavoring chemicals which impart flavors were more toxic than others. Based on flavoring chemical toxicity of the individual flavoring chemicals in e-liquids, flavors can be regulated. Further, our data indicate that tighter regulations are necessary to reduce the risk of inhalation toxicity due to exposure to e-liquids without nicotine and flavoring chemicals.

## Author contributions

TM, MP, KA, JG, IS, and IR: Conceived and designed the experiments; TM, MP, and KA: Performed the experiments and analyzed the data; TM, KA, and IR: wrote the manuscript.

### Conflict of interest statement

The authors declare that the research was conducted in the absence of any commercial or financial relationships that could be construed as a potential conflict of interest.
